# Viral suppression among HIV-positive patients on antiretroviral therapy in northwestern Nigeria: an eleven-year review of tertiary care centre records, January 2009–December 2019

**DOI:** 10.1186/s12879-021-06722-3

**Published:** 2021-10-02

**Authors:** Suleiman Bello Abdullahi, Olayinka Rasheed Ibrahim, Abdulkadir Baba Okeji, Rabilu Iliyasu Yandoma, Ibrahim Bashir, Suleiman Haladu, Suleiman Ahmad Idris, T. I. A. Oseni, Bello Muhammad Suleiman, Mohammed Yahaya, Mabel Kamweli Aworh, Mu’awiyyah Babale Sufiyan

**Affiliations:** 1 Department of Family Medicine, Federal Medical Centre, Murtala Mohammed Way (Jibia Bypass), P. M. B: 2121, Katsina, Nigeria; 2 Nigeria Field Epidemiology and Laboratory Training Programme, Abuja, Nigeria; 3 Department of Paediatrics, Federal Medical Centre, Katsina, Nigeria; 4 Department of Family Medicine, Federal Medical Centre, Lokoja, Nigeria; 5 Department of Haematology and Blood Transfusion, Federal Medical Centre, Katsina, Nigeria; 6 Department of Family Medicine, Ambrose Alli University, Ekpoma, Nigeria; 7 Department of Medical Microbiology and Parasitology, Usmanu Danfodiyo University, Sokoto, Nigeria; 8 Department of Community Medicine, Ahmadu Bello University Zaria, Kaduna, Nigeria

**Keywords:** Viral suppression, HIV-Positives, Antiretroviral Therapy, Nigeria

## Abstract

**Background:**

Human Immuno-Deficiency Virus (HIV) remains one of the world’s significant public health challenges. Viral suppression is the key indicator for treatment success in People living with HIV (PLHIV). We determined the level of viral suppression, and its associated factors among PLHIV attending Federal Medical Centre Katsina (FMC Katsina), Nigeria.

**Methods:**

This retrospective descriptive cross-sectional study was conducted on 913 HIV positive adults enrolled in care between January 2009 and December 2019. Information on socio-demographics, clinical, immunological, Viral load (VL), and other relevant parameters were extracted from the patients’ care records. The primary outcome was the proportion of patients that achieved viral suppression. We also analyzed variables that were associated with VL suppression.

**Results:**

Of 913, records of 831 (91.0%) registered patients were analyzed. During the period, 751 (90.4%) achieved viral suppression, 427 (51.4%) had CD4 counts  ≥ 500 and 477 (57.4%) were on HAART for ≥ 5 years. Majority, 793 (95.4%) were on first-line HAART regimen (Tenofovir-Lamivudine-Dolutegravir or Abacavir-Lamivudine-Dolutegravir), and 809 (97.4%) in the non-advanced stage (WHO stages 1 and 2). The median (interquartile range) of viral load was 20 (20–40) vs 19,989 (3311–110,340) cp/ml in virally suppressed, and unsuppressed  respectively. Factors associated with viral suppression included being unemployed (Adjusted OR [AOR] 4.9, 95% CI 2.771, 8.539), educated (AOR 4.2, 95% CI 1.098, 16.223), having a baseline CD4 count ≥ 500 cells/µl (AOR 2.7, 95% CI 1.588, 4.625), and being on first line HAART regimen [AOR 7.0, 95% CI 3.220, 15.648].

**Conclusions:**

Our study demonstrated a good viral suppression among PLHIV on HAART. Variables associated with viral suppression included unemployment, formal education, high baseline CD4 count, and first line HAART regimen.

## Background

Human Immuno-Deficiency Virus (HIV) is a leading cause of infections worldwide despite the success of highly active antiretroviral therapy (HAART) [1]. It remains one of the world’s most significant public health challenges, affecting about 38 million of the world’s population majority of whom are living in low and middle income countries [2]. Nigeria shares a portion of this estimate with a recent national prevalence of 1.4%. With varying prevalence across the country, Katsina state has the lowest prevalence of 0.3% while Akwa-Ibom state has the highest prevalence of 5.6% [3, 4].

The 90-90-90 target by UNAIDS envisages that by 2020, 90% of PLHIV will know their HIV status, 90% of people who know their HIV-positive status will be accessing treatment and 90% of people on treatment will have suppressed viral loads [5]. However, in 2018 Nigeria has only 67% of PLHIV that knew their status, of which 53% were on treatment and only 42% were virally suppressed [5]. The northwest zone of Nigeria also reflects similar statistics as only 9 out of 20 PLHIV on HAART achieved viral suppression [5, 6].

Viral load (VL) measurement is the gold standard for monitoring treatment success. PLHIV on HAART with unsuppressed viral load have a higher risk of disease progression, transmission, and mortality [7]. Available statistics have shown that Nigeria is far behind the target of 'UNAIDS 90-90-90 aspirations’ despite the recent downward trend in prevalence and incidence. Within the limits of our literature search on major databases- PubMed, Medline, Scopus, Google scholars, and Web of Science-there was no research that has evaluated viral suppression among HIV patients in the north-western part of Nigeria.

The objective of this study was to determine the level of viral suppression and its associated factors among HIV positive adults attending the HIV clinic at FMC Katsina, Nigeria.

## Methods

This retrospective cross-sectional descriptive study was conducted from January 2009 to December 2019 at FMC, Katsina. The hospital is a 1000 bed capacity, tertiary health facility. It serves as a referral center for both private and other public health facilities in the state, neighboring states, and the Niger Republic. The HIV clinic otherwise referred to as Action Aid clinic was established in 2006 and provides services for the diagnosis, treatment, and prevention of HIV, inclusive of free counseling and testing. It can accommodate about two hundred patients per clinic day and further subdivided into units. The HIV clinic units included the adult ART clinic, Paediatrics ART unit, the Prevention of Mother-to-Child Transmission (PMTCT) unit, HIV/TB co-infection unit, Pharmacy unit and the laboratory.

### Study participants and data collection

The source of data was from the database of the HIV clinic obtained from all PLHIV enrolled in ART care from January 2009 to December 2019 at FMC Katsina. A total of 1034 clients were registered within this period. The database provided information on socio-demographics, clinical, immunological, virological and other relevant parameters of patients. A total number of 913 adult clients were enrolled within the study period and they were all considered as the study subjects. We excluded children and adolescents less than 18 years on HAART. We used VL testing data for samples corresponding to HIV positive patients who had been on HAART for at least 6 months. Where there were more than one VL results, the most recent (not more than 12 months prior in line with the country’s national guideline for HIV) was used for the data. We abstracted data on VL testing results (for plasma) measured in terms of viral RNA copies/ml of blood. Before 2017, virological monitoring was carried out only in those with clinical suspicion of failure or poor adherence. Following policy change in the country and update of National guideline in 2018, all enrolled patients among the PLHIV had a minimum of yearly viral load monitoring. However, where the viral load was above the thresholds of 1000 copies/ml, intensified adherence counseling were carried out and viral load assays repeated within three to 6 months.

### Outcome measures

The primary outcome was virological suppression, defined as having ≤ 1000 copies of viral RNA/ml of blood for plasma, as provided by the National guideline for treatment and control of HIV/AIDS 2018. The secondary outcome of this study was factors associated with viral suppression.

### Definition of terms

Base line CD4 count: This was the CD4 count at the commencement of HAART.

Body mass index (BMI): The body mass index was determined by dividing the weight in kilogram with height in metre squared and classified using Centres for Disease Control (CDC) into underweight (< 18.5 kg/m^2^), normal or healthy weight (18.5 to 24.9 kg/m^2^), overweight (25 to 29.9 kg/m^2^) and obese (≥ 30 kg/m^2^).

TB status: This was defined as confirmation or diagnosis of Mycobacterium tuberculosis during the study past 1 year.

### Laboratory testing methods

The laboratory analysis was carried out by the CDC accredited laboratory of the Aminu Kano Teaching Hospital, Kano. For each sample collected from the patients, RNA was extracted and plasma VL determined using Polymerase chain reaction (PCR). The VL measurements were carried-out in 2018 and 2019.

### Data quality assurance

One of the authors extracted the data into Microsoft Excel spreadsheet and this was verified independently by two other co-authors to ensure accuracy and consistency of the data.

#### Data analysis

This was done using Epi-info version 7 and IBM SPSS statistics for Windows, version 25 (IBM Corp. Armonk, NY, USA). The proportion of subjects with virological suppression was expressed as percent. We compared the socio-demograhics (age, sex, educational status, marital status, employment, and occupation), BMI categories and distance from the health facility using chi-square and Fischer’s exact test where apporiate between the subjects that were virally suppressed and those without viral suppression. Besides, clinical and laboratory parameters (baseline CD4 count, duration on HAART, HAART regimen, WHO staging of HIV, and pregnancy status) were also compared between those that were virologically suppressed and unsuppressed subjects using Chi-square and Fischer’s exact test as appropriate. The viral loads were summarized as median with interquartile range (IQR) and compared using Man-Whitney U test between the two groups. Based on the literature, some variables (age, and gender) along with other variables (occupation, educational status, BMI categories, baseline CD4 count, duration on HAART, HAART regimens) with p-value less than 0.25 on the bivariate analysis were entered into binary logistic regression model to determine factors that were associated with virological suppression. The p-value for level of statistical significance was set at < 0.05.

## Results

**Fig. 1:**
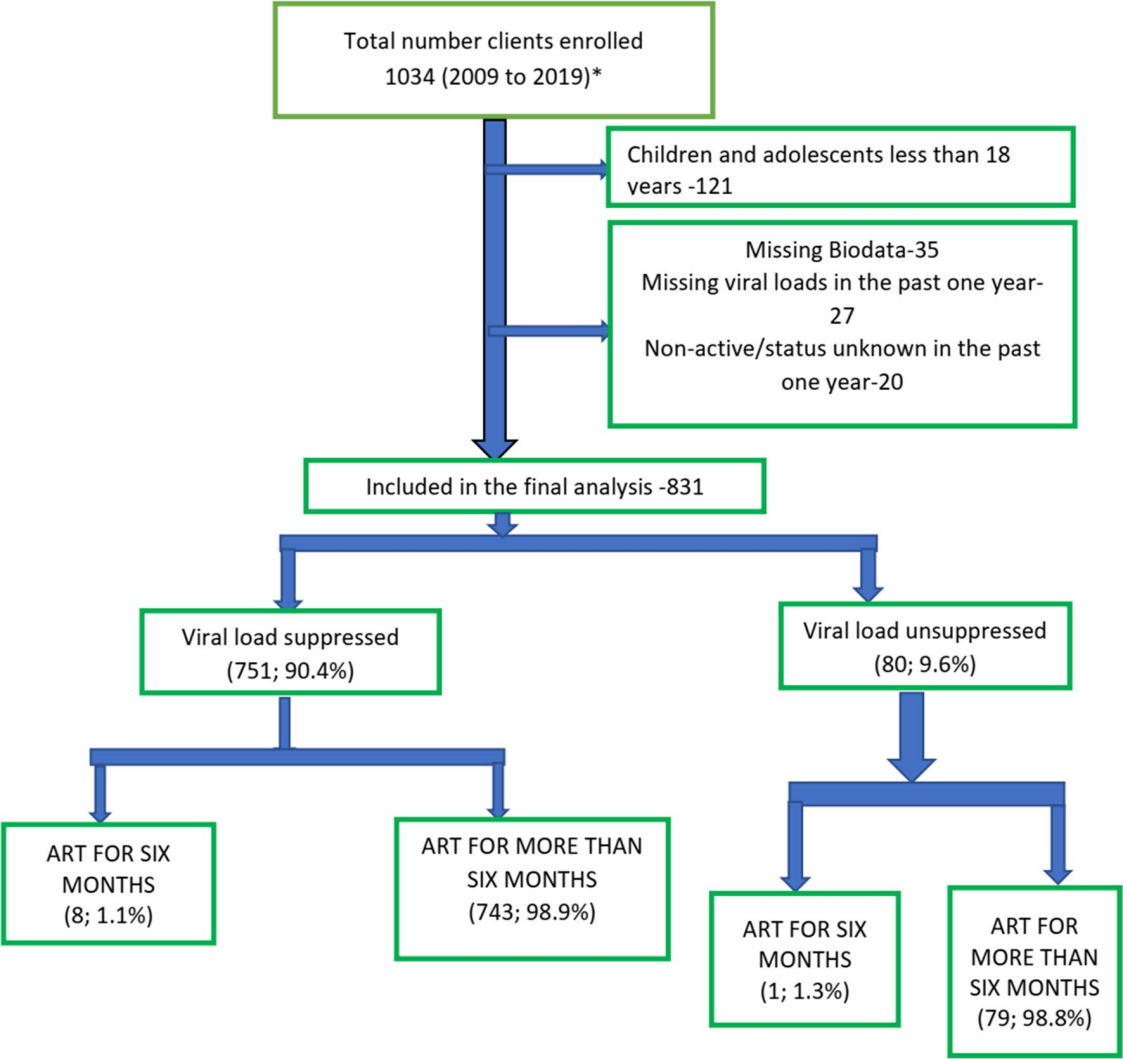
Flow chart of the study subjects. ART-Antiretroviral therapy; *all the subjects are on ART

During the study period, a total of 1034 subjects including children were enrolled into care at the centre. Out of the 1034 registered subjects, 913 (88.3%) were adults. Of the 913 adults, we excluded 82 subjects (35 had missing biodata, 27 had no traceable viral loads in the past 12 months, while the records of 20 showed non active status -no clinic visit and could not be traced in the past 1 year). Thus, we included 831 (91.0%) in the final data analysis as highlighted in Fig. 1.

The subjects age ranged from 18 to 77 years. The mean age (standard deviation) of the subjects was 34.5 ± 9.3 years. Most of the subjects were below the age of 50 years (782; 94.1%). There were more females, 578 (69.6%) than males. A total of 709 (85.3%) were unemployed while 78.6% were married as shown in Table 1.

**Table 1:** Socio-demographic profile of the study participants (n = 831)

Variables	Frequency (n)	Percentage (%)
Current age (years)		
18–27	56	6.7
28–37	295	35.5
38–47	305	36.7
48–57	138	16.6
58–67	34	4.1
68–77	2	0.2
≥ 78	1	0.1
Gender		
Male	253	30.4
Female	578	69.6
Occupation		
Business	7	0.8
Employed	114	13.7
Retired	1	0.12
Unemployed	709	85.3
Marital status		
Divorced	56	6.7
Married	653	78.6
Single	56	6.7
Widowed	66	7.9
Educational status		
Qur'anic	12	1.4
Primary	163	19.6
Secondary	536	64.5
Tertiary	104	12.5
None	16	1.9
Location		
Far from ^a^FMCK (≥ 100 km)	63	7.6
Near from FMCK (< 100 km)	768	92.4

^a^FMCK-Federal Medical Centre, Katsina

The median viral load was 20 (20–74) cp/ml in the study subjects. The median viral load in those that achieved viral suppression range from 20 to 925 cp/ml, while in those without viral suppression, VL range from 1170 to 2,468,443.0). In 2018, out of 87 subjects, 77 (88.5%) had viral suppression. Similarly, in 2019, out of 774 subjects, 704 (91.0%) had achieved viral suppression. The duration on HAART, TB status, WHO staging of the disease, and pregnancy status were not related to viral suppression. However, baseline CD4 count and HAART regimens were associated with viral suppression (Table 2).

**Table 2:** Factors associated with viral suppression (n = 831)

Variables	n (%)	Viral load suppressed n = 751	Viral load unsuppressed n = 80	P value
Baseline CD4 counts (cells/µl) *				
< 500	404 (48.6)	346 (46.1)	58 (72.5)	< 0.001
≥ 500	427 (51.4)	405 (53.9)	22 (27.5)	
Years on HAART				
< 5 years	354 (42.6)	325 (43.3)	29 (36.3)	0.237
≥ 5 years	477 (57.4)	426 (56.7)	51 (63.7)	
HAART Regimen				
First Line	793 (95.4)	728 (96.9)	65 (81.3)	< 0.001
Second Line	38 (4.6)	23 (3.1)	15 (18.7)	
TB confirmed**				
Yes	21 (2.5)	19 (2.8)	2 (2.8)	1.000f
No	731 (88.0)	662 (97.2)	69 (97.2)	
Missing	79 (9.5)			
Disease stage				
WHO stages 1 and 2	809 (97.4)	732 (97.5)	77 (96.3)	0.461f
WHO stages 3 and 4	22 (2.6)	19 (2.5)	3 (3.7)	
Pregnancy Status (n = 578)				
Not Pregnant	558 (96.5)	501 (96.7)	57 (95.0)	0.452f
Pregnant	20 (3.5)	17 (3.3)	3 (5.0)	
Viral Load (cp/ml) Median (IQR)	20 (20–74) Range (20 to 2,469,613)	20 (20–40) Range (20 to 925)	19,989 (3311 to 110,340) Range (1170 to 2,468,443.0)	< 0.001
2019 Viral Load (cp/ml) Median (IQR)	20 (20–70.5) Range (20 to 2,469,613) N = 744	20 (20–40) Range (20 to 845) N = 674	17,086.5 (3228.8–131,695 Range (1170 to 2,469,613) N = 70	< 0.001
2018 Viral Load (cp/ml) Median (IQR)	20 (20–119) Range (20 to 112,454) N = 87	20 (20–39.5) Range (20 to 925) N = 77	31,997.0 (4205.5–68,372.0) Range (1373 to 112,454) N = 10	< 0.001

*Baseline value of CD4 count at enrollment (Prior to the commencement of anti-retroviral therapy-ART); **TB confirmed cases among those with viral load suppression; IQR-Interquartile range; f-Fischer exact test

Tables 3 and 4 show the logistic regression analysis of factors that were associated with VL suppression. The age group and sex were not associated with viral load suppression. However, unemployed subjects had a significant association with viral suppression compared with employed subjects (Adjusted odds ratio [AOR] 4.86, 95% confidence interval 2.771 to 8.539). Similarly, subjects with formal education had a significant viral suppression compared with those without formal education(AOR 4.221, 95% CI 1.098, 16.223). A baseline CD4 count of 500 cells/µl or more was significantly associated with viral suppression (AOR 2.71, 95% CI 1.588, 4.625). Subjects on first line HAARTS also had significant viral suppression when compared with those on second line (7.208, 95% CI 3.220, 15.648).

**Table 3:** Unadjusted and adjusted binary logistic regression analysis of factors associated with Viral load suppression

Variables	Viral loads		Unadjusted			Adjusted		
	Suppressed (n = 751, %)	Unsuppressed (n = 80, %)	OR	95% CI	*p*	OR	95% CI	*P*
Age^a^								
18 to 35	258 (34.4)	29 (36.3)	1	0.606, 1.777	0.133	1	0.583, 1.906	0.861
36 to 45	277(36.9)	30 (37.5)	1.038			1.054		
46 and older	216 (28.8)	21 (26.3)	1.156	0.641, 2.086	0.207	1.116	0.534, 2.332	0.771
Gender								
Male	233 (31.0)	20 (25.0)	1	0.437, 1.258	0.267	1	0.358, 1.325	0.264
Female	518 (69.0)	60 (75.0)	0.741			0.689		
Occupation								
Employed	91 (12.1)	30 (37.5)	1	2.632, 7.196	< 0.001	1	2.771, 8.539	< 0.001
Unemployed	660 (87.9)	50 (62.5)	4.352			4.864		
Educational status								
No formal education	13 (1.7)	3 (3.8)	1	0.617, 7.932	0.223	1	1.098, 16.223	0.036
Formal education	738 (98.3)	77 (96.3)	2.212			4.221		
Marital status								
Unmarried	159 (21.2)	19 (23.8)	1	0.673, 1.998	0.593	–	–	–
Married	592 (78.8)	61 (76.3)	1.160					
BMI categories ≠								
18.5 to 24.9	289 (38.5)	39 (48.8)	1	1.032, 3.969	0.040	1.839	0.908, 3.726	0.091
< 18.5	180 (24.0)	12 (15.0)	2.024					
25 to 29.9	197 (26.2)	20 (25.0)	1.329	0.753, 2.347	0.327	1.385	0.748, 2.565	0.300
30 and above	85 (11.3)	9 (11.3)	1.275	0.594, 2.736	0.534	1.283	0.565, 2.917	0.551
Location^c^								
< 100 km	692 (92.1)	76 (95.0)	1	0.573, 4.583	0.363	–	–	–
≥ 100 km	59 (7.9)	4 (5.0)	1.620					

Variables included in the adjusted odds ratio were age, sex, occupation, educational status, BMI categories, baseline CD4 count, duration on HAART, and HAART regimens

^a^Age at the commencement of Anti-retroviral therapy; HAART-Highly active anti-retroviral therapy, Reg-regimen; BMI-Body mass index; CDC classification^≠^. ^c^Location of participants from FMC Katsina

**Table 4:** Binary logistic regression analysis of factors associated with Viral load suppression

Variables	Viral load		Unadjusted			Adjusted		
	Suppressed (n = 751, %)	Unsuppressed (80, %)	OR	95% CI	*p*	OR	95% CI	*p*
Baseline CD4 (cells/µl)								
< 500	346 (46.1)	58 (72.5)	1	1.851, 5.146	< 0.001	1	1.588, 4.625	< 0.001
≥ 500	405 (53.9)	22 (27.5)	3.086			2.710		
Years on HAART								
< 5	325 (43.3)	29 (36.3)	1	0.462, 1.202	0.228	1	0.686. 2.019	0.555
≥ 5	405 (56.7)	51 (63.7)	0.745			1.117		
HAART regimen								
Second line	23 (3.1)	15 (18.8)	1	3.634, 14.683	< 0.001	1	3.220,15.648	< 0.001
First line	728 (96.9)	65 (81.3)	7.304			7.208		
Disease stage								
WHO stage 1 & 2	732 (97.5)	77 (96.3)	1	0.193, 2.302	0.521	–	–	–
WHO stage 3 & 4	19 (2.5)	3 (3.8)	0.666					
Pregnancy status								
Pregnant (20)	17 (3.3)	3 (5.0)	1	0.441, 5.455	0.494	–	–	–
Not pregnant (558)	501 (96.7)	57 (95.0)	1.551					

Variables included in the adjusted odds ratio were age, sex, occupation, educational status, BMI categories, baseline CD4 count, duration on HAART, and HAART regimens

HAART-Highly active anti-retroviral therapy

## Discussion

The level of viral suppression in this study was high and it falls within the global target of 90% viral suppression among PLHIV on HAART by USAID [5]. This study also showed that the level of viral suppression in this cohort was one of the highest levels of viral suppression in low-middle income countries. This level is higher compared with the national level of viral suppression of 44.4% in the north-western part of Nigeria [6]. The value of viral suppression in this study was also higher compared to 79% reported in a multi-center Nigerian study [8], and 84% in Borno state, north-eastern Nigeria [9], 69% in Ghana [10] and 73% in northern Ethiopia [11]. High level of viral suppression in this study are comparable with the reports from Uganda where a level of 95% was observed for viral suppression after 12 months of HAART among PLHIV [12]. Similarly, the level of viral non-suppression obtained in this study is comparable to 9.0% and 7.0% reported in the African cohort study [13], and Vietnam [14] respectively. The high levels of viral suppression observed in this study compared to the other studies may be due to several reasons such as; the cut off value used for VL suppression in this study was VL < 1000 compared with a low value of 400 used in the earlier Nigerian studies that evaluated viral suppression after test and treat protocols [8] and after 6 months of initiation of first-line of HAART in a Moroccan study [15]. Furthermore, our centre is a tertiary health care centre with a dedicated unit for PLHIV and patients routinely undergo adherence counselling during their clinic visits which could have enhanced their compliance with their medications leading to viral suppression [16]. Our findings also suggest that the goal of achieving a 90% level of viral suppression is achievable in a resource-limited country like ours and the current approach in the management of HIV should be sustained.

This study also identified factors associated with viral suppression as having high baseline CD4 counts, being unemployed, being educated and being on first-line HAART. The study in Ethiopia reported a low baseline CD4 count and 2nd line HAART regimen to be associated with poor viral load suppression [11]. In contrast, the study in Borno State, north-eastern Nigeria found younger age group and marital status to be associated with poor viral suppression [9]. A South African study also identified low CD4 count to be associated with poor viral load suppression [17]. The CD4 cells are one of the prime targets of HIV, hence it’s fall corresponds to increasing viral loads. Although not the main marker of monitoring HIV, low CD4 counts at the beginning of treatment calls for closer monitoring of the PLHIV as they have a higher chance of having poor viral suppression.

Our study identified that being on the firstline line HAART was associated with good viral suppression when compared with those on the second-line. This is consistent with the observation in Borno State, northeast Nigeria where being on the second line HAARTs was associated with high viral load counts [9]. The African cohort study also found being on the second line HAART as predictive of viral suppression [13]. The higher level of non viral suppression among those on the second line HAART may indicate a high level of resistance as patients are moved to second-line following the failure of first-line. This is also a source of concern as non-viral suppression on second-line may necessitate a consideration for the commencement of third-line HAART which are presently not very available in resource limited countries like Nigeria. Besides, unemployed have higher odds of viral suppression with an AOR of 4.8. A study in Ghana that included occupations among factors that may predict non-suppression of viral load, did not observe any significant relationship [10]. Several factors may account for a higher level of suppression of viral load among the employed people in this study. The work schedules may affect their compliance and adherence levels. Also, those that work in the formal sectors may not be regular at clinic follow up due to their work schedules and the need for frequent permission for clinic attendance. Our study also showed that baseline CD4 counts greater or equal to 500 have higher odd of suppression of viral loads. Similarly, a study in Ethiopia also observed low CD4 count to be predictive of viral load non-suppression [18]. In Vietnam, low CD4 counts were also found to be predictive of non-viral suppression [14]. The findings of low baseline CD4 counts being associated with viral load non-suppression affirms the previous observation that patients with high viral loads tend to have low CD4 counts and suggests a slow viral clearance.

The strength of our study included the large number of subjects that had at least one viral load in the preceding 12 months. However, our study is limited being a retrospective study, as only few subjects had their viral load monitored before 2018. We also excluded few subjects (82) due to missing information. Although we compared with global set target of 90-90-90, the samples were not randomly drawn. Hence, our findings may not be generalizable. Furthermore, being a retrospective study, we could not adjust for some potential confounders, which may have affected our findings.

## Conclusion

Our study demonstrates a low level of viral non-suppression among PLHIV on HAART and the target of 90% viral suppression in the PLHIV on HAART by the USAID is achievable in resource constraint settings. Also, having a high baseline CD4 count, being employed, uneducated and being on first-line HAART are predictive of viral load suppression.

## Data Availability

The data that support the findings of this study are available from the Federal Medical Centre, Katsina HIV clinic. In the event that someone wants to request for the data for this study, he/she should seek permission from the Medical Director, Federal Medical Centre, Katsina in person of Dr SULEIMAN, Bello Muhammad (mbskt@yahoo.co.uk).

## Abbreviations

AIDS: Acquired Immune Deficiency Syndrome
AOR: Adjusted Odds Ratio
ART: Antiretroviral therapy
CD-4: Cluster of Differentiation 4
CDC: Centers for Disease Control and Prevention
CI: Confidence Interval
OR: Odds Ratio
HIV: Human Immunodeficiency Virus
PEPFAR: President’s Emergency Plan For AIDS Relief
PLHIV: People Living With HIV/AIDS
P-value: Probability value
UNAIDS: United Nations Programme on HIV and AIDS
VL: Viral Load
WHO: World Health Organization
FMC Katsina: Federal Medical Centre, Katsina

## References

[1] The Global Burden of Disease study (GBD 2015)_HIV collaborators. Estimate of global, regional, and national incidence, prevalence and mortality of HIV, 1980–2015: the global burden of disease study 2015. Lancet HIV 2016:3(8):e361–87. 10.1016/s2352-3018(16)30087-x. Accessed 22 Jan 2020. 10.1016/S2352-3018(16)30087-X PMC5056319 27470028

[2] World Health Organization. HIV/AIDS Fact File. https://www.who.int/news-room/fact-sheets/detail/hiv-aids. https://origins.who.int/features/factfiles/hiv/en. Accessed 1 Nov 2020.

[3] UNAIDS Press Release Abuja/Geneva 14th March 2019. https://www.unaids.org/en/resources/presscentre/pressreleaseandstatentarchive/2019/march/20190314_nigeria. Accessed 22 Jan 2020.

[4] National Agency for the Control of AIDS (NACA). Nigeria HIV Prevalence Rate. https://naca.gov.ng/nigeria-prevalence-rate/. Accessed 22 Jan 2020.

[5] UNAIDS. The 90–90–90 Ambitious Treatment Target to Help End AIDS Epidemic. http://www.unaids.org/en/resources/documents/2014/90-90-90. Accessed 22 Jan 2020]

[6] National Agency for the Control of AIDS (NACA). Nigeria HIV/AIDS Indicator and Impact Survey March 2019. Available at https://naca.gov.ng/wp-content/uploads/2019/03/NAIIS-Northwest. Accessed 22 Jan 2020.

[7] World Health Organization. What’s New in Treatment Monitoring: Viral Load and CD4 Testing. WHO-HIV-2017 Update July 2017. https://apps.who.int/iris/bitstream/handle/10665/235891/WHO-HIV-2017.22-eng. Accessed 20 Jan 2020.

[8] Stafford KA, Odafe SF, Lo J, Ibrahim R, Ehoche A, Niyang M (2019). Evaluation of the clinical outcomes of the *Test and Treat* strategy to implement *Treat All* in Nigeria: results from the Nigeria Multi-Center ART Study. PLoS ONE.

[9] Sunkanmi F, Paul Y, Peter D, Nsikan A, Joseph J, Opada E (2020). Factors influencing viral load non-suppression among people living with HIV (PLHIV) in Borno State, Nigeria: a case of Umaru Shehu Ultra-Modern Hospital. J Adv Med Res.

[10] Ofori-Attah P, Ameke LS, Obirikorang C, Orish VN, Kpene GE, Agboli E, et al.Viral suppression and its associated factors in hiv patients on highly active antiretroviral therapy (HAART): a retrospective study in the Ho Municipality, Ghana. AIDS Res Treat. 2020; 1–7.

[11] Desta AA, Tewolde WW, Futwi N, Gebrecherkos TG, Goyitom GG, Asfawosen AB (2020). HIV virological non-suppression and factors associated with non-suppression among adolescents and adults on antiretroviral therapy in northern Ethiopia: a retrospective study. BMC Infect Dis..

[12] Ssemwanga D, Asio J, Watera C, Nannyonjo M, Nassolo F, Lunkuse S (2020). Prevalence of viral load suppression, predictors of virological failure and patterns of HIV drug resistance after 12 and 48 months on first-line antiretroviral therapy: a national cross-sectional survey in Uganda. J Antimicrob Chemother.

[13] Kiweewa F, Esber A, Musingye E, Reed D, Crowell TA, Cham F (2019). HIV virologic failure and its predictors among HIV-infected adults on antiretroviral therapy in the African Cohort Study. PLoS One..

[14] Rangarajan S, Donn JC, Giang LT, Bui DD, Nguyen HH, Tou PB (2016). Factors associated with HIV viral load suppression on antiretroviral therapy in Vietnam. J Virus Era..

[15] Abebe G, Bonsa Z, Kebede W (2017). Treatment outcomes and associated factors in tuberculosis patients at Jimma University Medical Center: a 5-year retrospective study Gemeda. Int J Mycobacteriology..

[16] Mainaa EK, Mureithia H, Adana AA, Muriukib J, Lwembeb RM, Bukusi EA (2020). Incidences and factors associated with viral suppression or rebound among HIV patients on combination antiretroviral therapy from three counties in Kenya. Int J Infect Dis.

[17] Govender S, Otwombe K, Essien T, Panchia R, de Bruyn G, Mohapi L (2014). CD4 counts and viral loads of newly diagnosed hiv-infected individuals: implications for treatment as prevention. PLoS ONE.

[18] Bayu B, Tariku A, Bulti AB, Habitu YA, Derso T, Teshome DF (2017). Determinants of virological failure among patients on highly active antiretroviral therapy in University of Gondar Referral Hospital, Northwest Ethiopia: a case-control study. HIV/AIDS Res Palliat Care.

